# Spatial Risk Factors for Pillar 1 COVID‐19 Excess Cases and Mortality in Rural Eastern England, UK

**DOI:** 10.1111/risa.13835

**Published:** 2021-10-02

**Authors:** Julii Brainard, Steve Rushton, Tim Winters, Paul R. Hunter

**Affiliations:** ^1^ Norwich Medical School University of East Anglia Norwich UK; ^2^ School of Natural and Environmental Sciences Newcastle University Newcastle upon Tyne UK; ^3^ Insight and Analytics Norfolk County Council Norwich UK

**Keywords:** Ageing population, air quality, COVID‐19, deprivation, rurality

## Abstract

Understanding is still developing about spatial risk factors for COVID‐19 infection or mortality. This is a secondary analysis of patient records in a confined area of eastern England, covering persons who tested positive for SARS‐CoV‐2 through end May 2020, including dates of death and residence area. We obtained residence area data on air quality, deprivation levels, care home bed capacity, age distribution, rurality, access to employment centers, and population density. We considered these covariates as risk factors for excess cases and excess deaths in the 28 days after confirmation of positive Covid status relative to the overall case load and death recorded for the study area as a whole. We used the conditional autoregressive Besag—York–Mollie model to investigate the spatial dependency of cases and deaths allowing for a Poisson error structure. Structural equation models were applied to clarify relationships between predictors and outcomes. Excess case counts or excess deaths were both predicted by the percentage of population age 65 years, care home bed capacity and less rurality: older population and more urban areas saw excess cases. Greater deprivation did not correlate with excess case counts but was significantly linked to higher mortality rates after infection. Neither excess cases nor excess deaths were predicted by population density, travel time to local employment centers, or air quality indicators. Only 66% of mortality was explained by locally high case counts. Higher deprivation clearly linked to higher COVID‐19 mortality separate from wider community prevalence and other spatial risk factors.

## 1 INTRODUCTION

The respiratory illness COVID‐19 arises from infection with the SARS‐CoV‐2 virus. COVID‐19 is a still emerging disease that was first identified in early 2020. COVID‐19 was declared a Public Health Emergency of International Concern on 30 January 2020 (World Health Organization, 2020a) and reached pandemic status on 11 March 2020 (World Health Organization, 2020b). Economically damaging and socially disruptive measures have been implemented to control the outbreak in an effort to reduce excess mortality and health service demands linked to this disease (Anderson, Heesterbeek, Klinkenberg, & Hollingsworth, 2020).

In high‐income countries, COVID‐19 is thought to have a case fatality rate between 0.2% and 1.5% (Meyerowitz‐Katz & Merone, 2020; Rajgor, Lee, Archuleta, Bagdasarian, & Quek, 2020; Streeck et al., 2020). It was clear very early in the outbreak that disease severity was linked strongly to advanced patient age (European Centre for Disease Prevention and Control, 2020). COVID‐19 is usually a mild illness in children, is not commonly dangerous in adults under 50, and seems to only have mortality rates above 1% in adults above 50 years old (Onder, Rezza, & Brusaferro, 2020). Non‐Caucasian ethnicity is also clearly linked to higher hospitalization and mortality rates (Docherty et al., 2020). The importance of many other possible risk factors for severe disease outcomes is not as clearly established, however.

We aimed to explore possible associations between spatial variables and the risk of excess Covid cases or subsequent excess 28‐day mortality. The study design is a secondary analysis of a data source that described residents in a contained and mostly rural region of Eastern England who tested positive for SARS‐CoV‐2 using rt‐PCR. A not‐trivial proportion (21%, Affairs, 2020) of the population in England lives in predominantly rural areas. About half of the population in our study area lived in cities, the rest in small towns, town fringe, villages, or hamlets. It is valuable to document the risk factors for Covid infection or excess mortality in mixed or mostly rural localities, as the findings may differ from observations made about risk factors among overwhelmingly urban dwellers. Rural residents of England tend be older than city dwellers. Our study area had the distinction of having the most “elderly” population in Britain, with a median age of about 46 years old, which compares to a median age of 40.2 years for all U.K. residents in mid‐2018 (McCurdy, 2019). This older age profile suggested the population could be especially vulnerable during the Covid epidemic. Residential origin area was available for most of these patients which meant we could identify many of their spatial attributes. We used conditional autoregressive models in the Besag—York–Mollie framework to investigate whether there was spatial dependence in case detection or mortality following COVID‐19 positivity and the extent to which these could be linked to population density, socioeconomic deprivation, rurality, levels of air pollution, care home bed capacity, road network connectivity, and age demographics in residential areas.

## 2 METHODS

### 2.1 Data

The analysis covers patients who had a confirmed positive swab test by May 31, 2020 and for whom outcomes had been recorded by September 22, 2020. End of May approximately correlated with the end of the first “Wave” of the epidemic in England. A much later extraction date (September 22) than the census date (May 31) was desirable because we knew that it should adequately capture case counts and mortality, rather than have results biased by incomplete data due to delayed reporting.

The data set described Covid+ patients among the population of the English county of Norfolk and a single district (Waveney) in the adjacent county of Suffolk. For historical and geographical reasons, provision of health care in Norfolk and Waveney (N&W) is combined, currently under the commissioning powers held by the N&W clinical commissioning group (NWCCG). Norfolk and Waveney is a predominantly rural and coastal area in Eastern England, the United Kingdom, that extends roughly 55 by 40 miles. The population is approximately 1 million. The county is neither especially affluent nor deprived but does have areas among the 10% most and least deprived areas in England (Norfolk Insight, 2020). Appendix A1 shows percentiles statistics to indicate how representative Norfolk and Waveney are compared to other areas of England for air quality, deprivation, rurality, and driving times to nearby employment centers. Deaths among patients with COVID‐19 in Norfolk were already known to be strongly linked with advanced age, similar to data from other areas (see data for the single largest acute care provider in N&W in Appendix A2).

The data comprised individual COVID‐19 Pillar 1 positive test results for persons in the N&W area who received care from local National Health Service (NHS) trusts serving this population. Table I lists the NHS trusts who provided Pillar 1 records to NWCCG. Cases detected under the Pillar 1 framework were tested for possible COVID‐19 because of medical need for urgent treatment or occupational exposure (Department of Health and Social Care, 2020). During the study period (March–May 2020), access to Covid testing was severely limited in England (see policy history database held at https://www.health.org.uk/news‐and‐comment/charts‐and‐infographics/covid‐19‐policy‐tracker) which means that the vast majority of all detected cases were found under the Pillar 1 framework. The data set did not enable us to separate those tested for medical treatment needs from those with occupational exposure. We believe most of the records relate to persons with medical need, because 57% of the records were for persons age 65 or older (beyond the recent average age of retirement in England; Hofäcker, Schroeder, Li, & Flynn, 2016), and 75% of the records were for persons age 50+. The data were collected, cleaned, and provided to us by NWCCG. The data set reported which NHS trust identified need for testing, residence area, age, sex, hospital admission date (if applicable), discharge date where applicable, date that COVID‐positive swab was taken, and date of death when applicable. The data covered patients who tested positive for COVID‐19 in the period March 9–31 May, 2020. May 31 was chosen as the final date because it approximately coincides with the end of the first “wave” in Norfolk, and start of a three month period when local case count was relatively quite low. Fewer than 30 individual patients had multiple positive swab tests, on dates usually within 7 days of each other. We used only the earliest positive test date so that each individual appeared in the data set only once.

Four acute NHS hospital trusts regularly serve Norfolk and Waveney residents. Data available from NWCCG suggested that historically no more than 3% of NWCCG residents seek acute hospital care from NHS acute care providers not included in our analysis data set (see information in Appendix A3). All UK residents are entitled to attend any NHS hospital for acute care, but these data help to indicate that most Pillar 1 patients in N&W were likely to be captured in the local NHS trust records available to us. The northern and eastern sides of Norfolk as well as eastern side of Waveney adjoin with the North Sea so NHS care providers in the rest of England are relatively much less accessible (for N&W residents) than the health care providers that are listed in our data.

West Suffolk Hospital (WSH) was unique in our data set in never providing patient residence information. Omission of these WSH patients could lead to considerable underascertainment of COVID‐19 cases in areas that had many patients who visited WSH. About 14% of south Norfolk residents preferentially travel to WSH for urgent health care because of shorter driving distances compared to reaching alternative providers (presentation proportions in Appendix A3). Although residential location was missing from the WSH records, the West Suffolk data did list the patient's primary care provider. Most of the N&W cases linked to WSH (32/42, 76%) were registered with primary care providers in the south Norfolk town of Thetford. Therefore, we excluded the Thetford area (LSOA codes E010264:65‐78) from our analysis to better assess any correlations between COVID‐19 outcomes and spatial variables for the rest of N&W. The other 10 patients excluded from our analysis because their Pillar 1 test was obtained via contact with WSH were registered with nine different primary health care practices scattered across south and mid‐Norfolk.

Home residence for each Covid+ patient was resolved to lower super output area (LSOA). LSOAs are standard census units in England for which socioeconomic and other indicators have been calculated. LSOAs are designed to be fairly consistent in population but not geographic size. LSOAs in N&W ranged in size from 12 to 10,689 ha (median 129 ha, IQR 46–1062 ha). LSOAs typically each contain about 650 households (OCSI, n.d.), the 597 eligible LSOAs within N&W each had a median 1567 total residents (range 960–5685, IQR 1392–1856) in mid‐2019 (the most recent population estimates available). The information available or possible to derive at LSOA resolution include population density, population counts in specific age groups, rurality/urban characterization, deprivation domains, air quality indicators, transport connectivity. These data allowed us to test whether, for patients who tested positive for COVID‐19 and mortality within the subsequent 28 days in each LSOA area could be linked to any of these possible correlative factors, which are described in more detail below.

Counts of persons in specific age ranges in each LSOA was extracted using mid‐2019 estimates of persons registered by home address as recorded in general practice (GP) primary care records. These counts were available from the Office of National Statistics (www.ons.gov.uk/peoplepopulationandcommunity/populationandmigration/populationestimates/datasets/lowersuperoutputareamidyearpopulationestimatesnationalstatistics). The supplied data were available in single year bands, from 0 years old to age 90+. We used these data to calculate the percentage of resident population in each N&W LSOA that was age 65 years or older.

Total land area for each LSOA was available from geoportal.statistics.gov.uk/datasets. Combined with the population total in each LSOA, this information enabled us to calculate population density (resident persons/hectare), which we hypothesized might correlate with local physical and social contact rates, and that these contact rates might in turn relate to risk of excess cases.

A better indicator of local physical and social contact rates could be relative rurality. How rural or urban an LSOA was determined by using a classification scheme developed for the Department for the Environment, Food and Rural Affairs, updated to 2014 and available at https://www.geoportal.statistics.gov.uk (Bibby & Brindley, 2016). The classification schema devised by Bibby & Brindley relied on many decision rules to put areas into one of four categories (from most rural to most urban): hamlet, village, town, and fringe or urban depending on the population density, predominant land use in each LSOA and geographical context (such as distinguishing town edges from city edges). The data were available in four ordinal categories (1 = most rural to 4 = most urban) which were handled as a numeric value in the models.

Communities with higher social deprivation are recognized as having poorer health resilience and greater likelihood of relatively poor health outcomes (Cairns, Curtis, & Bambra, 2012). Relative deprivation in each LSOA was indicated by the Index of Multiple Deprivation 2019 (IMD2019, (McLennan et al., 2019)) available from https://www.gov.uk/government/statistics/english‐indices‐of‐deprivation‐2019. The IMD2019 is a nationally standardized ranking of relative deprivation encompassing many domains, available for all United Kingdom. The values were handled as raw ratio values ranging (within this data set) from 25 to 32,406. It is more conventional to use IMD2019 values as numeric values indicating relative decile. However, this was undesirable in this data set because data resolution would have been lost; most LSOAs in this study area are in IMD2019 deciles 3–7.

Air quality indicators were available for each LSOA as subdomain information within the IMD2019. The air quality measures were reported as concentration scores relative to (national standards for hazardous) reference thresholds (McLennan et al., 2019). Air quality was reported in five possible domains: NO_2_, SO_2_, benzene, particulates, and sum of the preceding four. Appendix A1 shows percentile distributions of air quality indicators (particulates and total scores) for N&W relative to the national distribution for all England. N&W LSOAs are fairly representative of national population exposures for SO_2_, benzene, and particulates. N&W has better total air quality and lower NO_2_. These values were highly intercorrelated with each other which made it inappropriate to put them all in one model. While SO_2_ concentrations varied little within N&W (Appendix A1), preliminary research has linked higher concentrations of fine particulate matter with higher COVID‐19 mortality in the United States (Wu, Nethery, Sabath, Braun, & Dominici, 2020). Therefore, we tried in the modeling only two of the air quality measures as potential correlates: particulate levels and total air quality score.

As of mid‐May 2020, Norfolk was one of the English counties with fewest COVID‐19 cases and deaths (Drury, 2020; Place, 2020). This low incidence was posited to relate to poor transport and infrastructure links to reach Norfolk from the rest of the United Kingdom. We hypothesized that poor transport links might also account for some variation in case counts between N&W LSOAs. Therefore, we obtained calculations of transport connectivity in 2017 available at LSOA level for the Department of Transport and supplied at www.gov.uk/government/statistical‐data‐sets/journey‐time‐statistics‐data‐tables‐jts#journey‐times‐connectivity‐jts09. The JTS09 data set report on many modes of transport but it was inappropriate to put more than one of the measures into a single model due to high multicollinearity. From this data set we tested only the variable “Travel time in minutes by car to nearest employment center with 500–4999 jobs” as a connectivity indicator because N&W is a predominantly rural area with a single main city (Norwich: mid‐2019 population estimate = 150,000) and many smaller market towns (typical population 2000–13,000). Private motor vehicles are known to be the main mode of transport in N&W, tending to account for more than 60% of journeys (Great Yarmouth Borough Council, 2019; Norfolk County Council, 2016).

During the first wave of COVID‐19 in England, it was widely acknowledged that a high proportion of deaths were among persons living in residential care homes (Burki, 2020). We obtained information about the total number of social care homes in each LSOA and their bed capacity from the Care Quality Commission (https://www.cqc.org.uk/about‐us/transparency/using‐cqc‐data). These counts were each also considered as potential predictors of COVID‐19 cases or deaths.

Notably, our data set does not contain information about ethnic profile of the population within each LSOA. Although such data are available within LSOAs, N&W is an area that is quite low in ethnic diversity, and this is even truer among persons who are most at risk of hospitalization or death from COVID‐19 (age 65+). Note that 96.5% of all‐age Norfolk residents self‐identified as “white” in the 2011 Census (Norfolk Insight, 2020). There was negligible utility in trying to test whether percentage BAME persons in residence areas might be predictive of Covid+ outcomes within this population. We view this omission as a strength as much as it is a limitation because relationships with correlates can be interpreted without uncertainty about whether minority ethnic demographics interacted differently with each other correlate.

### 2.2 Analysis

The spatial nature of the data meant that models had to account for possible spatial autocorrelation. Hence, we used the Besag–York–Mollie model (BYM) which is a conditional autoregressive model (Besag, York, & Mollié, 1991) to model the excess number of cases and deaths in LSOAs relative to those expected given the population size in each LSOA and total number of cases, deaths, and resident population of the entire study area. The BYM model is autoregressive in that it assesses the contribution of cases and deaths in neighboring LSOAs, to those recorded in individual LSOAs, based on geographical contiguity. The sociodemographic features for each LSOA were fixed effects in our models. We used these attributes as predictors in ecological regression to investigate their contribution to excess cases and excess deaths across the LSOAs, in comparison to the incidence of cases or deaths in the full study area. We analyzed excess cases and deaths with an initial model using all predictors and then refined the model to one in which all predictors had a significant impact on explaining risk. Variance inflation factors were calculated to confirm that coefficient confidence intervals were not excessively biased by multicollinearity.

To summarize, we calculated the expected number of cases for each LSOA to May 2020, given the population of the LSOA and the population of the whole study area and the total number of cases in all LSOAs. We then used a Besag–York–Mollie model with integrated nested Laplace approximation (INLA; https://www.r‐inla.org/what‐is‐inla) to compare observed number of cases with those expected to generate relative risk of cases and deaths for each LSOA. The null model with no covariates represents the relative risk of cases and deaths in LSOAs. Inclusion of covariates in this modeling framework is effectively an ecological regression which models the relative risk given the covariates included.

Preferred candidate models had the lowest deviance information criterion for the BYM intercept (Berg, Meyer, & Yu, 2004). Inclusion of variables in the final models was decided using the confidence intervals for parameter estimates (the regression coefficients). Variables for which the 95% confidence interval (95% CI) did not include zero were considered significant. A coefficient 95% CI above zero indicates significant prediction of excess deaths; below zero 95% CI would mean reduction in deaths compared to study area as a whole. Models were fit with a Poisson error structure. Models with zero‐inflated structure for count data (ZI Poisson models) were applied because they allow for structural effects. Ten percent of LSOAs had no cases, more than 60% had no deaths. The total variation explained by the spatial distribution of LSOAs was noted to check if there was spatial clustering. Analyses were undertaken in R using the INLA package.

Assessing the impacts of risk factors on incidence of disease is complicated by the fact that recording of cases of disease and the associated risk factors are measured at the same place geographically and are not therefore independent of each other. Risk factors may therefore interact or have indirect effects on disease. The regression analyses described above effectively assess the independent contribution of the individual covariates to risk, but do not account for indirect effects which may be important, but not assessed if the covariates are considered to be independent. To investigate possible relationships between covariates and their direct and indirect impacts on the number of cases of disease and numbers of deaths we used structural equation modeling (Rushton, Shirley, Sheridan, Lanyon, & O'Donnell, 2010). We hypothesized that the sociodemography of the population at risk depended on where people lived, with urban LSOAs having different population structure to those in rural areas. We created a conceptual model of the interactions between variables as a path diagram and then challenged the model with the observed data on disease incidence and mortality for the 597 LSOAs. We used a principle of parsimony, removing nonsignificant pathways from the model, to identify the suite of pathways that best represented relationships in the data, quantifying indirect pathways that impacted on both the number of cases and the number of deaths in LSOAs. Models were fit using the piecewise SEM package in R.

## 3 RESULTS

1977 unique individual patient records were eligible, that reported on confirmed swab tests by May 31, 2020 and who received their test result from contact with at least one of the eligible N&W health care trusts. Appendix A4 shows the procedure for subsetting eligible patients from the full data set supplied.

**Fig 1:**
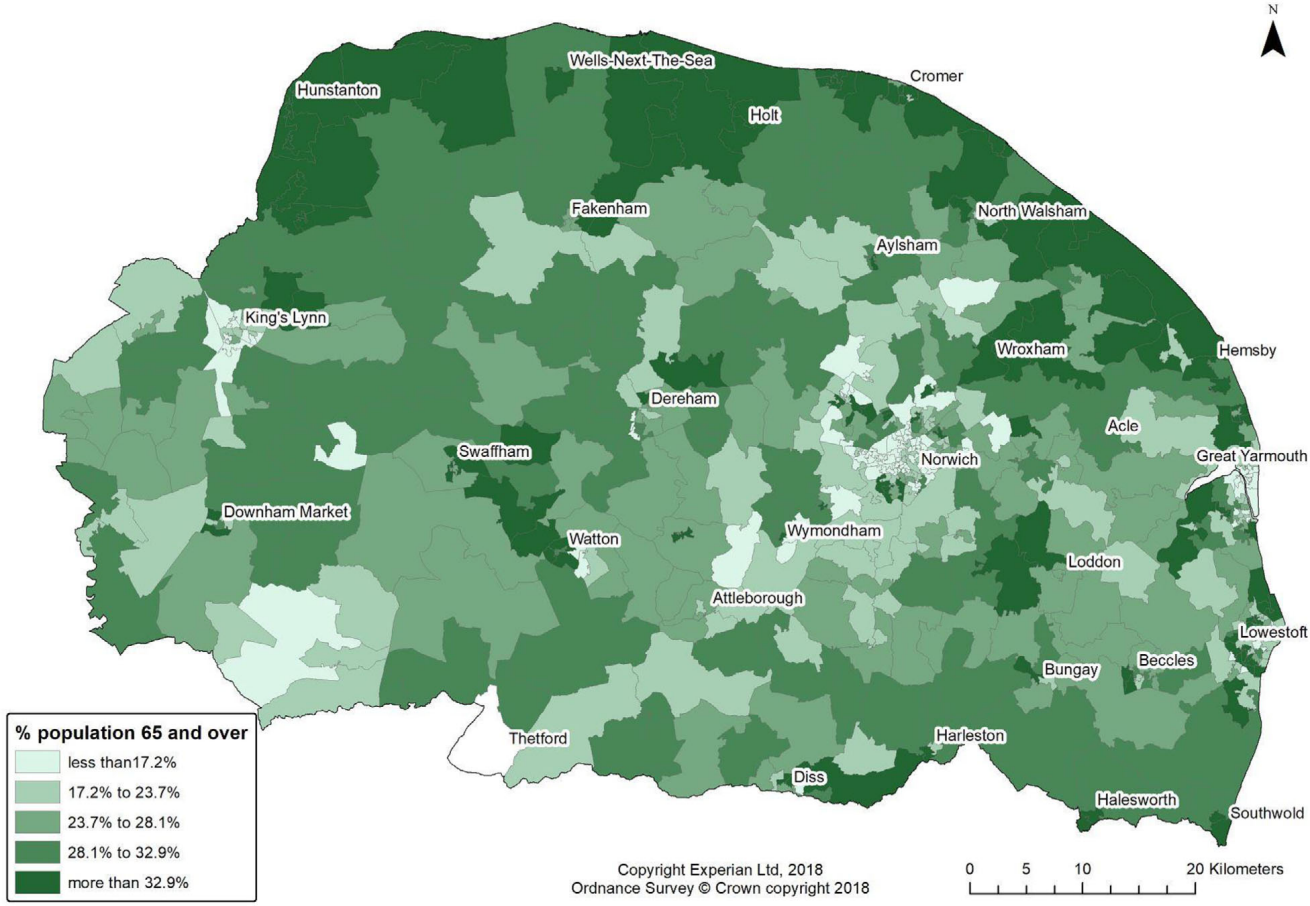
Proportion of population in each LSOA that was age 65 years and over.

**Fig 2:**
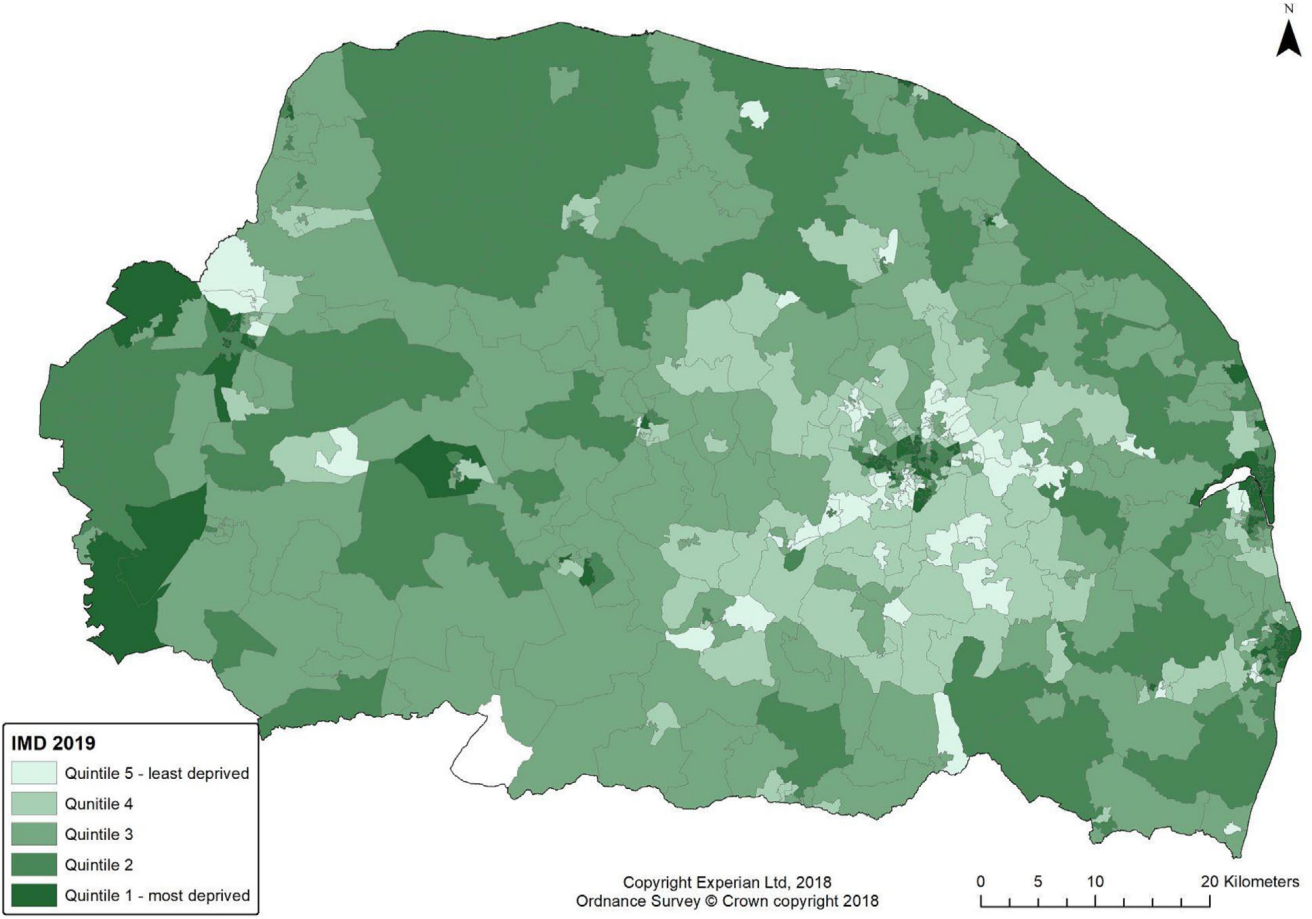
Index of Multiple Deprivation 2019 quintiles in study area.

Fig. 1 shows percent of resident population age 65+ for each LSOA mapped in quintiles with main town and city names, while Fig. 2 shows deprivation mapped in quintiles groups. In this study area, urban centers are relatively less prosperous and have a younger population than surrounding suburbs.

Results were as follows:

(i) Risk of excess cases

**Fig 3:**
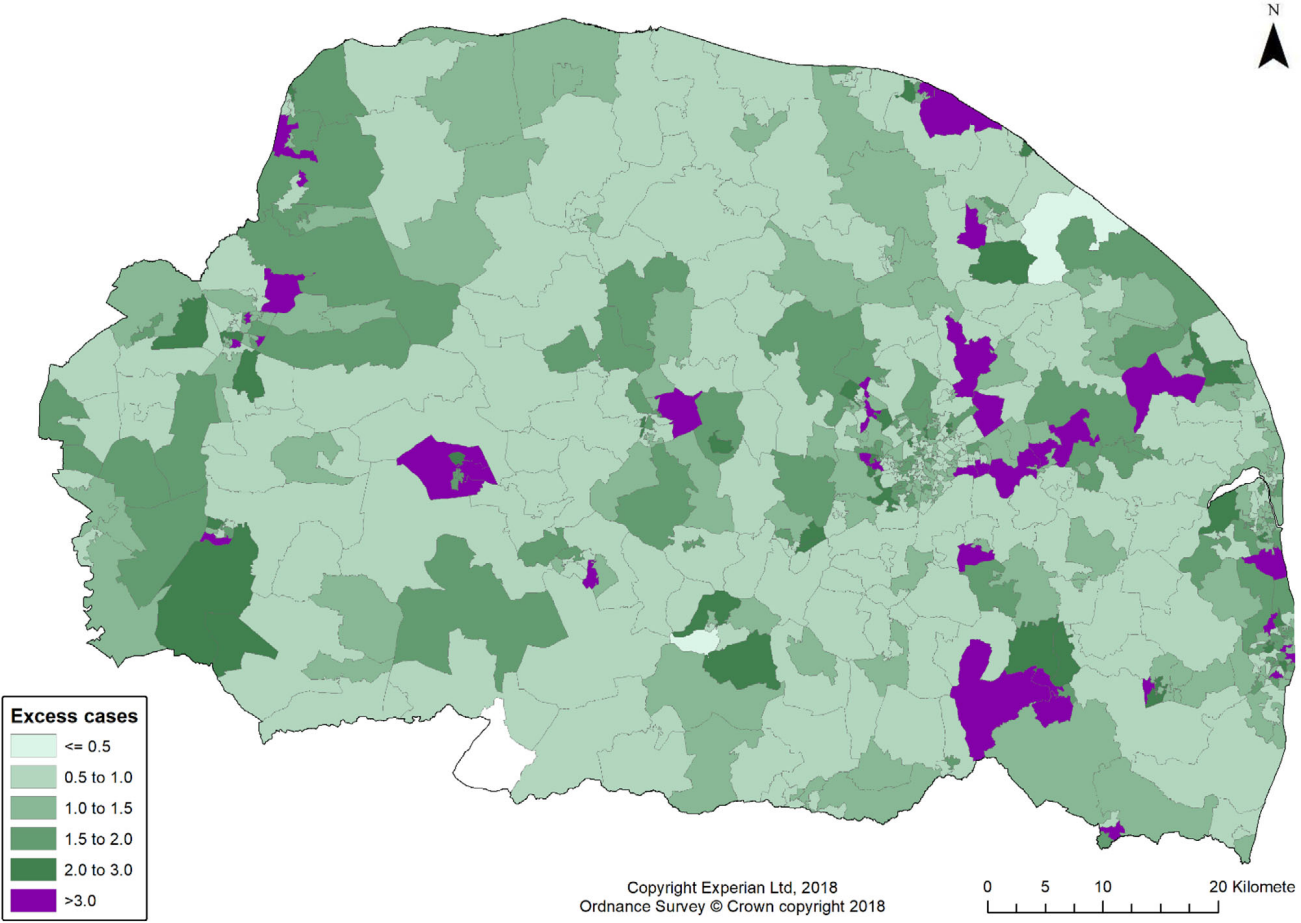
Excess case relative risk in study area.

Three variables significantly predicted excess cases of COVID‐19 in LSOAs. These were proportion of the LSOA resident population that was greater than 65 years of age (coefficient estimate = 1.193, 95% CI: 1.403–1.978) greater urbaneness (estimate 0.133, 95% CI: 0.053–0.214) and care home capacity (estimate 0.016, 95%CI 0.013–0.019). These indicate that aged populations in more urban areas or areas with more care homes had an excess of cases relative to LSOAs with younger populations in more rural settings. Analysis of the proportion of variation explained by the final model was low at 0.01 (on a scale of 0–1, where 1 is maximum possible variance explained by spatial distribution). Fig. 3 shows the relative risk (RR) of case emergence in the study area. RR = 1.0 means median risk (same as entire area). Relatively few areas had especially high risk. Areas with the highest excess risk of case emergence were widely dispersed, and both urban and rural, although arguably were more likely to be found in the eastern (more densely populated) third of the study area.

(ii) Risk of excess deaths

**Fig 4:**
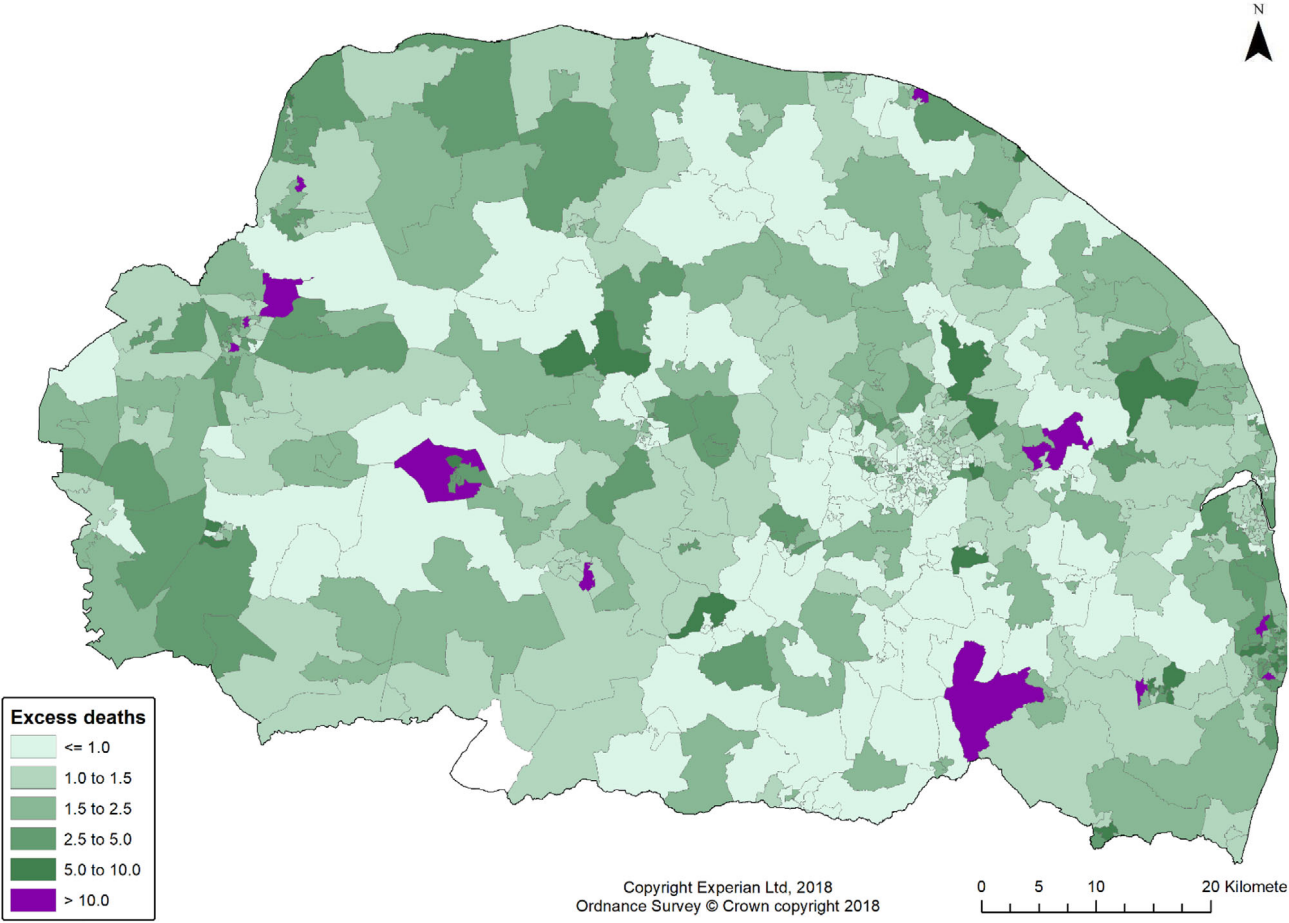
Excess death relative risk (following diagnosis) in study area.

Three variables were predictors of excess risk of dying after allowing for total cases in the same LSOA; the proportion of the population over 65 (estimate 3.632, 95%CI: 2.281–4.994), the care home bed capacity (estimate 0.028, 95%CI: 0.023–0.033) and lower deprivation for the LSOA (estimate −0.331, 95%CI:−0.506 to −0.160). This indicates that risk of dying was dependent on the proportion of the population over 65 and the extent of deprivation, with more deprived LSOAs having higher excess mortality, as well as local concentration of care home provision. The proportional contribution to the total variation explained by the spatial distribution of LSOAs was 0.02. Fig. 4 shows the relative risk (RR) of death following case diagnosis in the study area. RR < 1.0 means relatively lower risk, RR > 1.0 means higher than median risk. There were some areas with especially high risk of death (RR > 5 or even > 10) following diagnosis; this indicates greater variability in risk of excess death than there was variability in excess risk of case emergence.

Variance inflation factors for both models were all below 1.2, indicating collinearity did not bias model coefficients (Appendix A5).

### 3.1 Structural Equation Models

**Fig 5:**
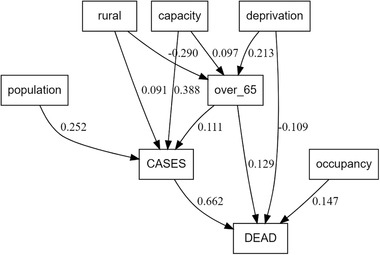
Summary statistics for structural equation (pathway) models.

Fig. 5 represents covariation between the candidate predictors and each other or the dependent variables used in our models, as obtained from SEMs. Table II shows the full model results. The numbers are the standard deviation change at the end of the arrow arising from one standard deviation change in the (origin) predictor. For example, a one standard deviation change in deprivation (IMD) leads to a 0.21 change in standard deviation change in the population percentage over age 65 variable. The greater the number, the stronger the covariation. These data indicate that increase in case counts explained 66% of the increase in mortality within LSOAs. There was clear indication that the covariates used in the conditional autoregressive model's analyses were not independent of each other and that the risk factors also contributed indirect effects to the level of disease and mortality. The percentage of the population age 65 years and older was especially likely to correlate with other spatially measured factors.

**Table I:** NHS Trusts That Provided Pillar 1 Test Results to NWCCG

**Acronym**	**Complete Name**	**Principle Types of Health Care Services**	**#cases start (final)**
ECCH	East Coast Community Health Care	Community hospitals that provide care procedures beyond remit of general practice	2 (1)
JPUH	James Paget University Hospital	Urgent, emergency, or consultant‐led secondary care	314 (306)
NNUH	Norfolk and Norwich University Hospital	Urgent, emergency, or consultant‐led secondary care	890 (870)
NSFT	Norfolk and Suffolk Foundation Trust	Mental health and dementia care services	16 (8)
NCHC	Norfolk Community Health and Care	Community hospitals that provide care procedures beyond remit of general practice	366 (336)
Other	Other NHS bodies	Mostly general practice primary care	392 (240)
QEH	Queen Elizabeth Hospital	Urgent, emergency, or consultant‐led secondary care	428 (347)
WSH	West Suffolk Hospital	Urgent, emergency, or consultant‐led secondary care	42 (0)

*Note*: #cases start refers to total cases in eligible monitoring period. #cases (final) means total in final analyzed data set. See data selection flowchart in Appendix A4. Some cases were linked to multiple NHS trusts so are counted twice in this table but not in the analyzed data set.

**Table II:** Selection of Structural Equation Model Coefficient Results

Response	Predictor	Estimate	Std. Error	DF	Critical Value	*p*‐Value	Std. Estimate
Cases	% age 65+	4.6864	1.6597	592	2.8236	0.0049	0.1111**
Cases	Rurality	0.3804	0.1579	592	2.4085	0.0163	0.0911*
Cases	∑ population	0.0021	0.0003	592	6.7509	0.0000	0.2519***
Cases	CH capacity	0.0777	0.0073	592	10.6997	0.0000	0.3881***
Died	Cases	0.2264	0.0101	592	22.4243	0.0000	0.6622***
Died	% age 65+	1.8601	0.4001	592	4.6493	0.0000	0.1290***
Died	IMD	−0.1830	0.0463	592	−3.9513	0.0001	0.1090***
Died	CH capacity	0.0099	0.0020	592	4.8828	0.0000	0.1447***
% age 65+	Rurality	−0.0287	0.0038	593	−7.6322	0.0000	0.2902***
% age 65+	CH capacity	0.0005	0.0002	593	2.5906	0.0098	0.0979**
% age 65+	IMD	0.0248	0.0044	593	5.6013	0.0000	0.2130***

*Note*: CH capacity = care home bed capacity. Died = Covid+ patients who died within 28 days of positive swab. DF = degrees of freedom. IMD = Index of Multiple Deprivation 2019. Std = standard. The rurality and IMD (deprivation) scales are such that low rank are more rural/more deprived. Significance thresholds (*p* <): * 0.05, ** 0.01, *** 0.001.

The SEM analyses show that the number of cases in an LSOA was significantly related to higher proportion of population over 65 and lack of rurality of the LSOA. However, the standardized coefficients were below 0.1 indicating that these parameters explained less than 10% of the variation in the number of cases. The number of cases was also strongly related to bed capacity in care homes in LSOAs, effectively representing the population at greatest risk. The number of deaths was strongly related to the total number of cases by end May, as well as greater proportion of the population over 65, higher deprivation and greater care home bed capacity.

## 4 DISCUSSION

Higher case counts in areas with a higher proportion of residents age 65+ probably reflects higher probability of severe disease (given that severity of disease is linked to advanced age). People tend to associate with persons close to their own age and a higher density of older adults may mean more social contacts among older adults with each other (Jackson & López‐Pintado, 2013). Other analyses have found higher mortality rates in areas with more older adults (Luo, Zhong, Sun, Wang, & White, 2021; Zhang & Schwartz, 2020). That worse outcomes (higher mortality) were linked to higher deprivation was also not surprising. A link between worse COVID‐19 outcomes and lower socioeconomic status has been reported in other COVID‐19 research (Berman et al., 2021; Lewis et al., 2020; Raisi‐Estabragh et al., 2020). That more socioeconomically deprived areas tend to suffer worse in pandemics may be a common feature of novel respiratory disease outbreaks, and was also a feature of the 2009 influenza pandemic in England and Wales (Rutter, Mytton, Mak, & Donaldson, 2012). This association may rise due to higher prevalence of key worker occupations, more barriers to social distancing, and overall lower health resilience in such communities (Cairns et al., 2012).

Finding increased mortality with less rurality may be due to greater mixing and expanded social contact networks for those who live within urban areas, and closer links to outside higher incidence areas. Other research on outcomes in the early pandemic phase found lower Covid infection or mortality rates in rural areas of the United States, even after adjustment for factors such as expected mortality rates based on local age demographics (Paul, Arif, Pokhrel, & Ghosh, 2021; Tian et al., 2021; Zhang & Schwartz, 2020). Lower mortality with increased rurality was also observed during the 1918–2019 influenza pandemic in England and Wales (Chowell, Bettencourt, Johnson, Alonso, & Viboud, 2008). However, higher rurality is an unreliable possible protection; arrival of pandemic conditions may only be delayed rather than prevented by rurality. For instance, the second wave of pandemic influenza affected rural areas much worse than the first outbreak wave had done in Wisconsin in 2009 (Truelove et al., 2011). Rural areas had lower case counts but higher mortality rates subsequent to infection in a study based on Georgia counties (Berman et al., 2021). In contrast to an opposite pattern in the spring of 2020, COVID‐19 case counts were observed to grow more quickly in rural than urban U.S. counties in the autumn of 2020 (Dobis & McGranahan, 2021).

Care home count and rurality had separate effects for case generation in our structural equation models. Similar to our findings, analysis in the United States found that counties with large care homes had higher COVID‐19 mortality rates than counties without large facilities (Kosar & Rahman, 2021; Luo et al., 2021). From registrations with the Care Quality Commission (https://www.cqc.org.uk/what‐we‐do/services‐we‐regulate/find‐care‐home) we observe that most N&W LSOAs (334/597) did not have any active registered care homes during March–May 2020, 189 LSOAs (32%) had just one care home while just 30 LSOAs (5%) had between three and seven operational care homes. The care home capacity variable was highly indicative of exactly where within suburbs, urban, or urban fringe areas the persons most likely to become Pillar 1 cases and experience subsequent Covid mortality would be found.

Negative findings (lack of correlation) in our models are useful results. Relative transport accessibility as indicated by the journey times measure did not emerge as significant predictor of cases or deaths in our models. Nor were air quality indicators predictors of case count or mortality. Population density had no separate effect on case count or deaths. These findings vary somewhat from other studies on rural populations; for instance Zhang and Schwartz (2020) found that higher population density was a key predictor of COVID‐19 positivity in a study of U.S. counties.

We note that the spatial variation in cases and deaths was negligible. There was little spatial dependency in the number of cases in LSOAs: neighboring LSOAs did not influence numbers of cases in adjacent LSOAs. Pillar 1 cases and subsequent deaths were not highly clustered at LSOA level geography. Cases and deaths seemed to occur independently of observable spatial contiguity.

### 4.1 Limitations

The findings relate very much to the types of cases that are found under the Pillar 1 testing framework. These are positive swabs found by testing health professionals (often found through surveillance rather than symptomatic presentation) and patients with urgent medical need. A more conventional sampling framework could include all symptomatic cases (including those without urgent medical needs). Some such cases were found concurrently in May 2020 under Pillar 2 testing protocols. Possible demographic differences between Pillar 1 and Pillar 2 cases in the county of Norfolk alone are described in the data shown as Appendix A5. Pillar 2 and Pillar 1 patients were not very different from each other in simple demographic traits (age distribution and sex).

Our analysis did not consider underlying health conditions. Individuals with multiple comorbidities, especially the metabolic syndrome triad (any three of unfavorable blood lipid profile, diabetes mellitus, hypertension, and obesity) seem to be at higher risk of mortality following Covid diagnosis (Ahlström et al., 2021; Li et al., 2021; Thakur et al., 2021). Our data set did not include any obesity or blood lipids information while specific health conditions were inconsistently recorded for the individual patients and could not be relied upon. We also have only incomplete information about the occupations of Covid‐positive patients in our data set; occupational risk may well have been more important than anything to do with residential origin for individual case status. However, most cases were above age 60 while most deceased were older than the statutory pension age (67 years currently). Occupational exposure is especially unlikely to be relevant to the mortality outcome.

We have tried to be transparent about the covariate specifications. We do not believe that different thresholds (such as considering population age 70 or older) would change the broad conclusions. Lack of variation in ethnic profile was both helpful and a drawback in the analysis. We cannot use data from this study region to test whether areas with larger ethnic minority populations had more cases or deaths. However, ethnicity not being an unobserved or poorly measured confounder makes it simpler to interpret the other candidate risk factors.

Our findings relate to new case emergence and mortality during a period when social distancing behavior in England varied greatly. Lockdown conditions started on 20 March 2020, with restrictions imposed to reduce social contact that were enforced by civil penalties (fines). The measures imposed were school closures for most pupils, bans on socializing with persons from outside one's own household or (size‐limited) mutual support group, closures of nonessential businesses and “stay‐at‐home” orders which proscribed loitering in public spaces (Hunter, Colon‐Gonzalez, Brainard, & Rushton, 2020). Some of these restrictions were modified or relaxed starting in May 2020. Because permitted types of social contact fluctuated, we cannot infer a great deal about specific social contact rules during this period and general spatial risk factors for case emergence or mortality following diagnosis.

We have not done sensitivity analysis to explore potential variability in the results dependent on geographical units used (the modifiable areal unit problem; Fotheringham & Wong, 1991). We would not expect the broad conclusions and relationships to greatly change had we used different geographical units of approximately the same population size and geographic extent as those used here. Use of coarser geographical resolution (larger origin areas) would probably lead to weaker relationships between covariates and outcomes in our models.

We made a key simplifying assumption about area connectivity in our spatial autoregressive models. The assumption was that transmission was mostly likely between LSOAs with a common border due to proximity and physical contact rates. This seems especially less likely to be true with respect to care home proximity to other care homes. In reality, virus spread between care homes could happen due to a shared delivery services, health care professional visitors, staff or administration teams that worked in geographically disparate locations. Genomic sequencing in March–August 2020 found a Covid lineage in many Norfolk care homes that was absent in the wider community (Quadram Institute, 2020), suggesting either shared origin disease introduction events and/or transmission between multiple care homes. We had no specific information on visitor, supplier, staff, or management working across multiple premises, which would be needed to define more realistic linkages between care homes. We acknowledge that our neighborhood definition was conservative. We also may have underestimated other spatial features of the pandemic that we could not quantify.

## 5 CONCLUSIONS

The number of cases in LSOAs were clearly dependent on the age demographic of the populations and their lack of ruralness. Older profile and less rural areas had more cases and more deaths. Allowing for the local age structure and rurality, sociodemographic status of the LSOA was linked with deaths from COVID‐19, but not full incidence of Pillar 1 cases in the wider study region. The results indicate that deprivation was an important predictor of poor outcomes subsequent to infection during the early part of the Covid epidemic in rural England.

## Supporting information


**Appendix A1**. LSOA percentile variables for numeric variables used as predictors in models (local values with national comparisons).
**Appendix A2**. Demographic traits of patients who died at NNUH.
**Appendix A3**. Emergency admissions of Norfolk & Waveney resident patients to acute care providers, aggregated by each of the five constituent clinical commissioning group areas for financial year April 2019‐ March 2020.
**Appendix A4**. Selection procedure for eligible records.
**Appendix A5**. Variance inflation factors for models fit as linear regressions, to test for problematic multi‐collinearity that might unduly bias 95% confidence intervals on coefficient estimates.
**Appendix A6**. Comparison of demographics of persons in Pillar 1 and Pillar 2 testing frameworks, through 6 August 2020.
