# Supplementary material for: Spatial Risk Factors for Pillar 1 COVID‐19 Excess Cases and Mortality in Rural Eastern England, UK
Source: Risk Anal. 2021 Oct 2;42(7):1571–84. doi: 10.1111/risa.13835 (PMC8661982; doi:10.1111/risa.13835)
Supplement: Supplementary file 1 — Appendix A1. LSOA percentile variables for numeric variables used as predictors in models (local values with national comparisons). Appendix A2. Demographic traits of patients who died at NNUH. Appendix A3. Emergency admissions of Norfolk & Waveney resident patients to acute care providers, aggregated by each of the five constituent clinical commissioning group areas for financial year April 2019‐ March 2020. Appendix A4. Selection procedure for eligible records. Appendix A5. Variance inflation factors for models fit as linear regressions, to test for problematic multi‐collinearity that might unduly bias 95% confidence intervals on coefficient estimates. Appendix A6. Comparison of demographics of persons in Pillar 1 and Pillar 2 testing frameworks, through 6 August 2020. [file RISA-42-1571-s001.docx]

**APPENDICES: SUPPORTING INFORMATION**

**Appendix A1.** This table shows the LSOA percentile values for numeric variables (ratios of local values to hazardous thresholds). Norfolk & Waveney area compared to all England, or the %s within the rural/urban categories. N&W LSOAs are fairly representative of national population exposures for SO_2_, Benzenes and particulate levels. N&W has better total air quality and lower NO_2_. N&W LSOAs rate as slightly more deprived than the national profile; N&W has slightly longer driving times to reach employment centres. N&W is much more rural than most of England, which results in lower population density and less air pollution.

| **What** | **Where** | **5%** | **25%** | **50%** | **75%** | **95%** |
| --- | --- | --- | --- | --- | --- | --- |
| AirQual | England | 0.54 | 0.71 | 0.84 | 0.97 | 1.17 |
| AirQual | N&W | 0.65 | 0.695 | 0.75 | 0.86 | 1.01 |
|  |  |  |  |  |  |  |
| SO2 | England | 0.02 | 0.02 | 0.03 | 0.03 | 0.05 |
| SO2 | N&W | 0.02 | 0.02 | 0.02 | 0.03 | 0.04 |
|  |  |  |  |  |  |  |
| Benzene | England | 0.04 | 0.06 | 0.08 | 0.1 | 0.13 |
| Benzene | N&W | 0.05 | 0.06 | 0.07 | 0.09 | 0.12 |
|  |  |  |  |  |  |  |
| NO2 | England | 0.19 | 0.27 | 0.34 | 0.42 | 0.56 |
| NO2 | N&W | 0.22 | 0.24 | 0.28 | 0.35 | 0.45 |
|  |  |  |  |  |  |  |
| Partics | England | 0.28 | 0.36 | 0.39 | 0.42 | 0.46 |
| Partics | N&W | 0.36 | 0.37 | 0.38 | 0.4 | 0.42 |

| **What** | **Where** | **5%** | **25%** | **50%** | **75%** | **95%** |
| --- | --- | --- | --- | --- | --- | --- |
| IMD2019 | England | 3951 | 12821 | 20003 | 26667 | 31681 |
| IMD2019 | N&W | 2489 | 10461 | 16044 | 22459 | 30080 |

| **What** | | **Where** | | **Hamlet** | | **Village** | | **Town/Fringe** | | **Urban > 10k** | | |
| --- | --- | --- | --- | --- | --- | --- | --- | --- | --- | --- | --- | --- |
| % rurality category | | England | | 3.3% | | 6.9% | | 8.6% | | 75.7% | |  |
| As above | | N&W | | 4.0% | | 26.7% | | 20.7% | | 48.6% | |  |
|  |  | |  | |  | |  | |  | | |  |
| **What** | **Where** | | **5%** | | **25%** | | **50%** | | **75%** | | **95%** | |
| Drive time | England | | 6.29 | | 7.13 | | 8.11 | | 9.67 | | 13.32 | |
| Drive time | N&W | | 6.3 | | 7.27 | | 8.54 | | 11.17 | | 14.85 | |

Note: Drive time (units = minutes) to reach an employment centre with at least 500 jobs. AirQual refers to air quality unified domain raw score in the Index of Multiple Deprivation 2019.

**Appendix A2.** The patients who died were 58% male, 42% female. 1.7% did not have underlying conditions. Deaths may have occurred > 28 days after first +swab test. Deaths occurred in the period 24 March – 30 June 2020. Data come from Norfolk and Norwich University Hospital (NNUH) only. The NNUH is the single largest facility providing acute health care services to the N&W population (see supporting data in **Appendix** A3). 86% of deaths were among persons age 70+. Source: Data published by NNUH at <http://www.nnuh.nhs.uk/news/2020/05/daily-announcement-covid-19-deaths/>.

**Appendix A3.** Emergency admissions of Norfolk & Waveney resident patients to acute care providers, aggregated by each of the five constituent clinical commissioning group areas for financial year April 2019- March 2020. Source: Jon Fox of NWCCG, data held as of 22.5.2020.

| **CCG** | **NNUH** | **JPUH** | **QEH** | **WSH** | **Other** |
| --- | --- | --- | --- | --- | --- |
| **North** | 83.8% | 4.8% | 9.6% | 0.1% | 1.7% |
| **Norwich** | 97.0% | 0.5% | 0.2% | 0.1% | 2.2% |
| **South** | 81.6% | 0.8% | 1.1% | 14.0% | 2.6% |
| **West** | 3.7% | 0.1% | 92.0% | 0.9% | 3.3% |
| **GY&W** | 10.2% | 87.0% | 0.1% | 0.1% | 2.6% |

**Notes**: CCGs are North = North Norfolk, Norwich, South = South Norfolk, West = West Norfolk, GY&W = Great Yarmouth & Waveney. Acute care centres are NNUH = Norfolk & Norwich University Hospital, JPUH = James Paget University Hospital (GY&W area), QEH = Queen Elizabeth Hospital (Kings Lynn, West Norfolk) and WSH = West Suffolk Hospital (Bury St. Edmunds, just south of the county of Norfolk).

**Appendix A4.**  Selection procedure for eligible records

Dataset was supplied on 22 September 2020, n=2490 unique patient records.

130 records removed because earliest COVID+ test was after 31 May

2360 records in monitoring period

42 records removed because only linked to West Suffolk provider

2318 records

228 records without LSOA code, 13 records from ineligible LSOAs

1977 records for patients resident in 516 of potential total 597 eligible N&W LSOAs. 424 of these patients died within 28 days of +swab.

**Appendix A5.** Variance inflation factors for models fit as linear regressions, to test for problematic multi-collinearity that might unduly bias 95% confidence intervals on coefficient estimates.

Cases

| Variable | VIF | 1/VIF |
| --- | --- | --- |
|  |  |  |
| % population age 65+ years | 1.14 | 0.876 |
| Urban rank (higher is more urban) | 1.11 | 0.897 |
| Care home bed capacity | 1.03 | 0.972 |
|  |  |  |
| Mean VIF | 1.09 |  |

Deaths

| Variable | VIF | 1/VIF |
| --- | --- | --- |
|  |  |  |
| % population age 65+ years | 1.09 | 0.917 |
| Deprivation rank (higher is less deprived) | 1.06 | 0.939 |
| Care home bed capacity | 1.02 | 0.976 |
|  |  |  |
| Mean VIF | 1.06 |  |

**Appendix A6**. Comparison of demographics of persons in Pillar 1 and Pillar 2 testing frameworks, through 6 August 2020. We include these data to address whether Pillar 2 patients might be very different from Pillar 1. Pillar 2 data were only for residents of the county of Norfolk (we do not have Pillar 2 data for the district of Waveney). The data show some differences in demographic profile of cases found under Pillar 1 vs. Pillar 2 test frameworks.

By 6 August, Fewer persons had tested positive under the Pillar 2 framework than under Pillar 1 sampling frame. The percentage of female persons who tested positive under either Pillar 1 or Pillar 2 were fairly similar. However, males were more likely to be Pillar 1 patients at age 50+. Persons age 50-59 or age 80+ were more likely to be tested under Pillar 1 than Pillar 2. Persons under 20 yrs old were especially likely to be tested under Pillar 2.
